# Abnormalities of Skeletal Muscle, Adipocyte Tissue, and Lipid Metabolism in Heart Failure: Practical Therapeutic Targets

**DOI:** 10.3389/fcvm.2020.00079

**Published:** 2020-05-12

**Authors:** Shingo Takada, Hisataka Sabe, Shintaro Kinugawa

**Affiliations:** ^1^Faculty of Lifelong Sport, Department of Sports Education, Hokusho University, Ebetsu, Japan; ^2^Department of Molecular Biology, Hokkaido University Graduate School of Medicine, Sapporo, Japan; ^3^Department of Cardiovascular Medicine, Hokkaido University Graduate School of Medicine, Sapporo, Japan

**Keywords:** mitochondria, ectopic fat, sarcopenia/cachexia, peripheral blood mononuclear cell, myokine, exercise

## Abstract

Chronic diseases, including heart failure (HF), are often accompanied with skeletal muscle abnormalities in both quality and quantity, which are the major cause of impairment of the activities of daily living and quality of life. We have shown that skeletal muscle abnormalities are a hallmark of HF, in which metabolic pathways involving phosphocreatine and fatty acids are largely affected. Not only in HF, but the dysfunction of fatty acid metabolism may also occur in many chronic diseases, such as arteriosclerosis, as well as through insufficient physical exercise. Decreased fatty acid catabolism affects adenosine triphosphate (ATP) production in mitochondria, via decreased activity of the tricarboxylic acid cycle; and may cause abnormal accumulation of adipose tissue accompanied with hyperoxidation and ectopic lipid deposition. Such impairments of lipid metabolism are in turn detrimental to skeletal muscle, which is hence a chicken-and-egg problem between skeletal muscle and HF. In this review, we first discuss skeletal muscle abnormalities in HF, including sarcopenia; particularly their association with lipid metabolism and adipose tissue. On the other hand, the precise mechanisms involved in metabolic reprogramming and dysfunction are beginning to be understood, and an imbalance of daily nutritional intake of individuals has been found to be a causative factor for the development and worsening of HF. Physical exercise has long been known to be beneficial for the prevention and even treatment of HF. Again, the molecular mechanisms by which exercise promotes skeletal muscle as well as cardiac muscle functions are being clarified by recent studies. We propose that it is now the time to develop more “natural” methods to prevent and treat HF, rather than merely relying on drugs and medical interventions. Further analysis of the basic design of and molecular mechanisms involved in the human body, particularly the inextricable association between physical exercise and the integrity and functional plasticity of skeletal and cardiac muscles is required.

## Introduction

**Figure 1:**
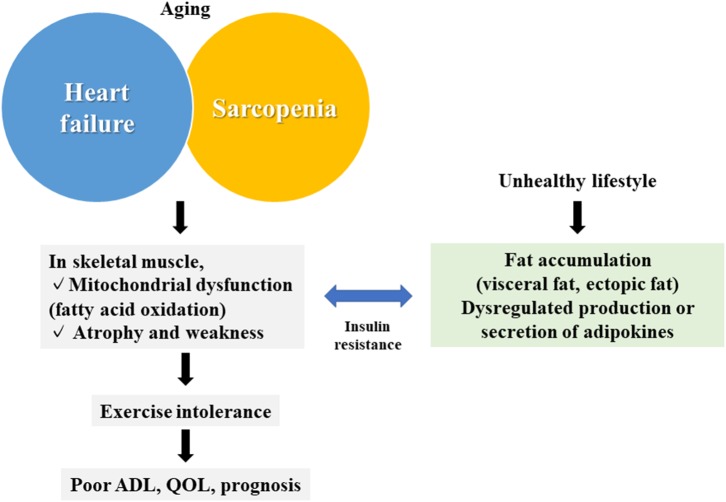
Summary of the association between skeletal muscle abnormalities and heart failure. Skeletal muscle abnormalities (mitochondrial dysfunction and muscular atrophy/muscular weakness) in heart failure and sarcopenia are associated with fat accumulation and abnormalities of adipokines caused by an unhealthy lifestyle, leading to reduced exercise tolerance, and a poor prognosis. QOL, quality of life; ADL, activities of daily living.

Chronic diseases, particularly heart failure (HF), cause qualitative and quantitative abnormalities, not only in the target organ but also in distant organs, such as the skeletal muscle (1, 2). Skeletal muscle abnormalities impair the activities of daily living and the quality of life, which are major causes of the impairment of exercise tolerance and the poor prognosis of patients with HF (1). However, circulatory failure is often followed by hypoperfusion, hypoxia, inflammation, and oxidative stress, and even by an imbalance of metabolic catabolism/anabolism, aggravated muscle wasting, including cachexia, and the activation of some neurohumoral factors (1–5). Sarcopenia is also a skeletal muscle disease (6). Patients with HF often have sarcopenia, in which HF and sarcopenia cooperatively worsen skeletal muscle conditions (7) (Figure 1).

The skeletal muscle utilizes glycolysis (anaerobic) and mitochondria (aerobic) to generate adenosine triphosphate (ATP). Our research group previously demonstrated that skeletal muscle mitochondria are the major target of HF, and mitochondrial dysfunction may lead to an accumulation of lipids in the skeletal muscle (8–10). Abnormal accumulation of adipocytes then affect whole body condition (11). In this review, we first summarize our current knowledge on the abnormalities of skeletal muscle and adipose tissue in HF and sarcopenia; and then discuss possible methods to prevent and/or treat such abnormalities, as well as the exercise intolerance observed in HF.

## Abnormalities of Skeletal Muscle in HF

ATP is the energy source for the contraction of skeletal muscle. However, only small amounts of ATP are present in the cytoplasm of skeletal muscle cells in the steady state (~8 mmol/kg wet weight of muscle). Needless to mention, the Embden-Myerhof glycolysis pathway generates ATP from ADP anaerobically. Skeletal muscle is rich in mitochondria, which produce ATP from ADP aerobically via oxidative phosphorylation (OXPHOS) (12, 13). On the other hand, phosphocreatine (PCr) serves as a rapidly mobilizable reserve in the skeletal muscle, as well as in the brain, to regenerate ATP from ADP anaerobically. PCr is generated from creatine by its phosphorylation, which requires ATP.

Using a cycle ergometer based on magnetic resonance spectroscopy (MRS), our research group previously measured energy metabolism of the quadriceps muscles of patients with chronic HF (unless patients did not have severe circulatory dysfunction) and healthy individuals (14, 15). We found that PCr, but not ATP, in the skeletal muscle becomes almost depleted during maximal systemic exercise both in patients and normal subjects. However, rates of the PCr decrease were significantly more rapid in patients with HF than in normal individuals, in which rates of PCr exhaustion correlated with the impairment of exercise tolerance (peak oxygen uptake and anaerobic threshold) of patients. Our results indicated that patients with HF may have impaired metabolism in their skeletal muscles, which primarily affects PCr exhaustion. The molecular bases of this rapid PCr exhaustion remains to be clarified; i.e., whether it is caused by dysfunction of PCr/creatine metabolism on its own or by insufficient production of ATP either anaerobically (glycolysis) or aerobically (mitochondrial OXPHOS). The identification of such information will then provide novel therapeutic targets of HF.

Skeletal muscle atrophy and weakness also occur in patients with HF, and correlates with their poor prognosis (16, 17). Chronic HF is generally associated with enhanced metabolic catabolism and reduced anabolism (18–26). For example, an increase in blood levels of catabolic hormones (cortisol, catecholamines, and angiotensin II) and a decrease in anabolic hormones (dehydroepiandrosterone sulfate, testosterone, and insulin-like growth factor I) have been shown to be closely associated with muscle atrophy, disease severity, and the poor prognosis of patients (18–20, 22). In particular, testosterone is tightly linked to exercise tolerance (22), and the efficacy of testosterone replacement therapy has been proven in various clinical trials. However, as testosterone often shows side effects, nonsteroidal androgen receptor agonists are being studied (27, 28). On the other hand, in a mouse HF model, molecules of the ubiquitin-proteasome system (atrogin 1 and muscle RING-finger protein-1) were shown to be upregulated and to cause further muscle atrophy (29). Moreover, skeletal muscle cell death is enhanced in patients with HF (29). Together with the above issues, therefore, both the qualitative (metabolic impairment) and quantitative (muscular atrophy) abnormalities of the skeletal muscle are hallmarks of patients with HF (Figure 1).

## Sarcopenia and Cachexia in HF

Sarcopenia was previously defined as a “syndrome” in 2010 (30), but is now recognized as a “skeletal muscle disease” (6). On the other hand, the diagnostic criteria of the Asian Sarcopenia Working Group includes lean limb mass and lean skeletal muscle mass (31). Furthermore, in the European Society of Cardiology guidelines for the diagnosis and treatment of acute and chronic HF, sarcopenia is defined as follows (32).

“Skeletal muscle wasting, when associated with impaired mobility and symptoms (termed sarcopenia or myopenia), occurs in 30–50% of patients with the HF reduced ejection fraction (HFrEF) (33). In its most severe form, it is associated with frailty and poor morbidity and mortality (34). Potential treatments may include appetite stimulants, exercise training (35), and anabolic agents, including testosterone, in combination with the application of nutritional supplements, and anti-catabolic interventions, although none is of proven benefit and their safety is unknown” (36).

Sarcopenia worsens HF (7). Pathologically, there is a crucial difference between HF and sarcopenia, i.e., HF results in a decrease in slow muscle fibers (oxidative fibers), whereas sarcopenia results in a decrease in fast muscle fibers (fast glycolytic fibers). Moreover, sarcopenia lacks any early diagnostic markers (37). In a study of 200 patients with HF in which 39 patients (19.5%) had sarcopenia, oxygen uptake (exercise capacity), 6-min walking distance, left ventricular ejection fraction, grip strength, and quadriceps strength were all significantly lower in the patients with sarcopenia than in those without sarcopenia (33). Another study also suggested a higher risk of mortality in patients with HF with sarcopenia, by further reducing the peak oxygen uptake (38). On the other hand, cachexia is accompanied by a loss of body weight (fat, skeletal muscle, and bone tissue), loss of appetite, and loss of body composition and function (39). Cachexia thus shares significant similarities with sarcopenia, and can be considered as a secondary sarcopenia. It was reported that there is a loss of skeletal muscle mass without a loss of body weight (sarcopenia) in early HF, which is often followed by a loss of body weight (cachexia) (40). Therefore, the occurrence of cachexia in patients with HF is a biomarker predictive for an increase in severity of chronic HF (16). However, the molecular links among sarcopenia, cachexia, and HF, with regard to the precise molecular/biochemical mechanisms, still remain largely elusive (Figure 1).

## Adipose Tissues and Fatty Acids in HF

### White Adipose Tissue (WAT) and Brown Adipose Tissue (BAT)

Adipose tissues are crucial in regulating cardiovascular functions, primarily through the secretion of adipocytokines controlling the cardiovascular endocrine and paracrine systems (11).

WAT is an endocrine organ that secretes adipokines, besides its role as an energy store (41, 42). When obesity occurs, it often triggers the secretion of adipokines, as well as induces inflammatory responses, leading to insulin resistance of the skeletal muscle and liver (11). Our previous studies have shown that patients with obesity or metabolic syndrome demonstrate an accumulation of visceral fats and white adipocytes, as well as systemic insulin resistance and the dysfunction of skeletal muscle mitochondria (10, 43–49). Intriguingly, patients with chronic HF and mouse HF models also demonstrate systemic insulin resistance (50–52). However, the molecular details as to how systemic insulin resistance often occurs in patients with HF still await to be clarified.

Shimizu et al. (53–55) have recently demonstrated that chronic inflammation in visceral fat causes systemic insulin resistance and exacerbates HF. They showed that visceral fat inflammation in patients with HF is caused by enhanced sympathetic nerve signaling, excessive fat melting, the production of reactive oxygen species (ROS), and the DNA damage-induced activation of the p53-nuclear factor-kappa B pathway. Blockade of this pathway suppressed visceral fat inflammation and improved systemic insulin resistance and cardiac function. Moreover, systemic insulin resistance may also be associated with mitochondrial dysfunction in the skeletal muscle (43–46, 48, 49, 56).

BAT is important for body heat production (57). However, recent studies demonstrated the more profound roles of BAT as a center controlling glucose and lipid metabolism of the whole body (58–63). On the other hand, impairment of BAT in obesity increases the number of lipid droplets and reduces the amount of mitochondria in BAT cells (i.e., “whitening” of BAT) (64). In contrast, when vascular endothelial growth factor α is expressed in the whitening BAT, its “rebrowning” occurs, which helps to improve insulin resistance of the whole body (64). These changes are reversible (64). Furthermore, low body temperature is an independent marker of the poor outcome of patients with HF and a reduced EF (65). Moreover, a decrease in body temperature predicts the time of rehospitalization and poor survival rate (66). Therefore, BAT impairment is also crucial to the worsening of HF, for which the precise molecular mechanisms await to be clarified.

### Ectopic Fat Deposition in the Skeletal Muscle of Patients With HF

Mechanisms by which cardiovascular diseases progress include the loss of metabolic balance between glycolipid synthesis and energy consumption in insulin-sensitive organs (67). On the other hand, the ectopic deposition of fats, which is causative of cardiovascular diseases, may also occur regardless of glucose/insulin tolerance (68). Moreover, ectopic fat deposition in tissues other than the heart (e.g., pericardial fats), including skeletal muscle, is also thought to be involved in HF pathology (11, 69) (Figure 1).

Reduced fatty acid oxidation in skeletal muscle mitochondria was observed in HF animal models (70–72). Using proton MRS, we measured intramyocellular lipid (IMCL) levels; and found that it is significantly increased in the skeletal muscle of patients with HF compared with healthy subjects, and is hence closely associated with lowered skeletal muscle energy metabolism and reduced whole-body exercise tolerance (8). Consistently, levels of three-hydroxyacyl-CoA dehydrogenase, a key enzyme of β-oxidation, were significantly reduced in the skeletal muscle of patients with HF and of an animal model (70, 71, 73, 74). Such skeletal muscle abnormalities of HF mice were shown to associate with systemic insulin resistance (50–52). Likewise, the amounts of IMCL in skeletal muscles were associated with insulin resistance in type 2 diabetic patients (75). In this disease, exercise therapy and diet therapy reduced lipid droplet levels and improved insulin resistance (43). Therefore, adequate exercise and a proper diet appear to be an efficient method to treat HF and associated insulin resistance (Figure 1).

## Targeting Skeletal Muscle Abnormalities in HF Therapy

The following are possible interventions for patients with HF via lifestyle improvement (exercise/diet), drugs (exercise mimetics), and proteins/peptides, to improve the integrity of their skeletal muscle and adipose tissue.

### Lifestyle Interventions

#### Exercise Training

Continuous exercise training, including cardiac rehabilitation, has many beneficial effects to treat heart diseases (76). Endurance or resistance training of patients with HF is recommended by the American Heart Association and the Japanese Circulation Society (77, 78). Recently, moderate to high intensity interval training has been studied as an exercise prescription of HF (79, 80), as alternatives to anaerobic (about 90% of the maximum heart rate) and/or aerobic exercises (81). Elucidation of these effects and mechanisms will help further develop more precise exercise prescriptions for HF.

#### Lipids, Particularly Linoleic Acid

Higher levels of plasma eicosapentaenoic acid, an essential omega-3 fatty acid, is closely associated with a reduced risk of HF, with both reduced and preserved EF (82). On the other hand, the combined supplementation of l-alanyl-l-glutamine and polyunsaturated fatty acids has been shown not to improve exercise performance or muscle function (83). Linoleic acid is an essential omega-6 fatty acid and is the major fatty acid moiety of cardiolipin, which is central to the assembly of mitochondrial OXPHOS components. We found that cardiolipin content in cardiac mitochondria becomes significantly lower in HF model mice, to be consistent with lowered OXPHOS activities (84). We thus fed HF model mice with a relatively high amount of linoleic acid (~6 mg/kg body weight/day) in their daily food for 4 weeks; and found that such an amount of daily intake of linoleic acid improves both mitochondrial and cardiac functions of HF mice, in which the assembly of the complex II subunits and the complex III_2_/complex IV supercomplex of mitochondrial OXPHOS were improved. Consistent with our results, a recent prospective study also demonstrated that a high linoleic acid concentration in blood is associated with a lower risk of HF incidence in aged men (85). On the other hand, it should be noted that an excess intake of linoleic acid is well-known to be harmful to health, such as by causing arteriosclerosis (86). However, ironically, we found that among the different fatty acids, only the dietary intake of linoleic acid is significantly lower in New York Heart Association (NYHA) class III patients compared with class II patients (84).

### Interventions Using a Device or Surgery

Cardiac resynchronization therapy, a left ventricular assist device, and percutaneous transluminal mitral commissurotomy, ameliorate hemodynamics immediately after surgery for HF, but do not immediately improve exercise intolerance and take longer than exercise training (87–89). This suggests that skeletal muscle abnormalities mediate hemodynamics and exercise tolerance, and further studies should be performed to clarify this point in the future.

### Drug Treatment Using Exercise Mimetics

#### Available Pharmacological Treatments

##### Renin-angiotensin system (RAS) inhibitors

Angiotensin-converting enzyme inhibitors, angiotensin receptor blockers (ARBs), antihypertensive drugs can help improve skeletal muscle abnormalities and the exercise intolerance of patients with HF (90). We have shown previously that excessive ROS production induced by activating RAS in skeletal muscle is a possible mechanism causing skeletal muscle abnormalities in HF mice (50–52); and demonstrated that (pro)renin receptor inhibitors, a decoy peptide of the handle region of mouse (pro)renin, and ARBs can improve insulin resistance, including in the skeletal muscle of HF mice (50–52). We also showed that the administration of angiotensin II, the main effector molecule of the RAS, induces similar skeletal muscle abnormalities to HF, which can be improved by inhibiting NAD(P)H-derived ROS production (91, 92).

##### Dipeptidyl peptidase 4 (DPP-4) inhibitor and glucagon-like peptide-1 (GLP-1)

Treatment with the incretin hormone GLP-1 (93) was reported to improve exercise intolerance of patients with HF (94). We analyzed whether the DPP-4 inhibitor, which increases GLP-1 levels (95), as well as an agonist against the GLP-1 receptor, improves skeletal muscle abnormalities and exercise intolerance in HF model mice. Our results indicated that these drugs are effective in improving mitochondrial biogenesis in the skeletal muscle of HF mice, in which lowered exercise capacity and mitochondrial function/volume, and altered fiber types of skeletal muscles (a shift toward the fast-twitch fiber type) are restored to a certain extent, without notably affecting infarct size and cardiac function (74).

##### Sodium-glucose cotransporter 2 (SGLT2) inhibitors

SGLT2 is a transporter that is expressed in the proximal tubules of the kidneys and reabsorbs ~90% of glomerular filtered glucose. SGLT2 inhibitors promote urinary glucose excretion, resulting in hypoglycemic and weight-loss effects independent of insulin action. The SGLT2 inhibitor ipragliflozin improved fatty liver syndrome of *ob*/*ob* obese mice fed a high-fat diet (96). This suggests that the increased energy storage capacity of adipocytes may be associated with the suppression of ectopic fat accumulation in the liver. On the other hand, treatment with an SGLT2 inhibitor was shown to alleviate disease progression, including the death of patients with HF with a reduced EF regardless of the presence or absence of diabetes (97). Our recent study showed that the SGLT2 inhibitor empagliflozin improves fatty acid oxidation in skeletal muscle mitochondria of HF mice (71). However, molecular links between SGLT2 and HF still remain largely elusive.

### Endocrine Factors

#### Myokines

Epidemiological studies have shown that continuous physical exercise exerts a variety of medical benefits throughout the body and contributes to prolonging the lifespan of an individual (98). More than 10 years ago, it was reported that various hormone-like bioactive substances are secreted from the skeletal muscle. Pedersen et al. (99) suggested that cytokines and other peptides that are produced, expressed, and released by muscle fibers and exert either paracrine or endocrine effects should be classified as “myokines.” Very interestingly, brain-derived neurotrophic factor (BDNF) is secreted from the skeletal muscle upon physical exercise and is thus categorized as a myokine (100). We found that serum levels of BDNF were significantly lower in patients with HF than healthy subjects (101). Univariate analysis demonstrated a significant positive correlation between serum BDNF levels and peak VO_2_ (oxygen uptake) among all study subjects, including patients with HF (101). By multivariate analysis, peak VO_2_ was identified as an independent determinant of serum BDNF level (99). We have moreover shown that low levels of serum BDNF statistically correlate with the poor outcomes of patients with HF (102). Decreased skeletal muscle BDNF correlated with decreased exercise capacity in HF model mice (70). We designed a treatment to improve the impaired exercise capacity as well as the dysfunction of skeletal muscle mitochondria of myocardial infarction (MI) mice. At 2 weeks after inducing MI, we divided mice into two groups: one was treated with recombinant human BDNF (rhBDNF) by daily subcutaneous injections for an additional 2 weeks, and the other was injected with empty vehicle. At 4 weeks, (i.e., after 2 weeks of treatment), we found that the rhBDNF-treated MI mice demonstrated improved cardiac mitochondrial respiration and exercise intolerance compared with control MI mice (70). Molecularly, rhBDNF increased mitochondrial biogenesis and fatty acid oxidation via the upregulation of AMP-activated protein kinase (AMPK) α-peroxisome proliferator-activated receptor γ coactivator 1α (PGC-1α) signaling in skeletal muscle (70). Our studies were the first to show that BDNF expression is decreased in the skeletal muscle of HF mice after the induction of MI, and that rhBDNF improves exercise capacity and skeletal muscle mitochondrial dysfunction of the HF mice.

Apelin is another myokine produced by skeletal muscle upon physical exercise that is beneficial against exercise tolerance (103). Serum levels of apelin are also reduced in an age-dependent manner in humans and rodents. Mice deficient in either apelin or its receptor demonstrated substantial alterations in muscle function during aging. Molecularly, apelin promotes mitochondrial biogenesis, autophagy, and anti-inflammatory responses of myofibers, and also enhances the regeneration potential of muscle stem cells. Therefore, similar to BDNF, apelin can be used to diagnose early sarcopenia, and provides an excellent therapeutic target to prevent age-associated muscle weakness (103).

Myonectin is a myokine that is upregulated in skeletal muscle and blood by exercise (104). Myonectin is an endurance exercise-induced myokine that ameliorates acute myocardial ischemic injury by suppressing apoptosis and inflammation in the heart (105). Likewise, BDNF protects against cardiac dysfunction after MI (106). Thus, these data suggest that myonectin and BDNF are myokines that mediate the beneficial actions of exercise on cardiovascular health.

#### Adipokines

Adiponectin is an antidiabetic adipokine. Its receptors possess a seven-transmembrane topology with the amino terminus located intracellularly, which is opposite to that of G-protein-coupled receptors. Iwabu et al. provided lines of evidence that adiponectin induces extracellular Ca^2+^ influx via adiponectin receptor 1 (AdipoR1), which is necessary for the subsequent activation of Ca^2+^/calmodulin-dependent protein kinase kinase b (CaMKKb), AMPK, and sirtuin1 (SIRT1), the increase in expression and decrease in acetylation of PGC-1α, and increase in myocyte mitochondrial number. Moreover, muscle-specific disruption of AdipoR1 suppressed the adiponectin-mediated increase in intracellular Ca^2+^ concentration, and decreased the activation of CaMKK, AMPK, and SIRT1 by adiponectin. The suppression of AdipoR1 also resulted in a decrease in PGC-1α expression and deacetylation, decrease in mitochondrial content, and decrease in oxidative type I fibers, and a decrease in oxidative stress-detoxifying enzymes in skeletal muscle, which were associated with insulin resistance and decreased exercise endurance. Decreased levels of adiponectin and AdipoR1 in obesity may have causative roles in the mitochondrial dysfunction and insulin resistance observed in diabetes (107).

Paralogs of adiponectin, such as C1q/tumor necrosis factor-related protein 9 in adipokines protect against acute cardiac damage in response to pathological stimuli (e.g., lipopolysaccharides) and myocardial ischemia-reperfusion, by suppressing inflammation through the AdipoR1/AMPK-dependent pathway (108, 109). Therefore, adiponectin in particular has protective effects, in not only skeletal muscle but also in the heart.

#### Hepatokines

It is well-known that the responsiveness to physical exercise differs markedly among different people. “Exercise resistance” is considered to be congenital, with no evidence of acquired causative factors. The antioxidative hepatokine selenoprotein P (SeP) was shown to cause exercise resistance through its muscle receptor, low-density lipoprotein receptor-related protein 1 (LRP1) (110). SeP-deficient mice showed a “super-endurance” phenotype after exercise training, which was accompanied by enhanced ROS production, AMPK phosphorylation, and PGC-1α expression in skeletal muscle. Supplementation with the antioxidant N-acetylcysteine reduced ROS production and endurance capacity in SeP-deficient mice. SeP treatment impaired hydrogen peroxide-induced adaptations through LRP1 in cultured myotubes and suppressed exercise-induced AMPK phosphorylation and *Ppargc1a* gene expression in mouse skeletal muscle, and these effects were inhibited in mice with muscle-specific LRP1 deficiency. Furthermore, increased amounts of circulating SeP predicted the ineffectiveness of training on endurance capacity in humans. Thus, it was proposed that inhibitors of the SeP-LRP1 axis may function as exercise-enhancing drugs for the treatment of diseases associated with a sedentary lifestyle (110).

#### Osteokines

Aerobic exercise was recently shown to increase blood levels of osteocalcin, which is produced by osteoblasts, in mice and humans, whereas blood osteocalcin decreases during aging in mice, monkeys, and humans (111). On the other hand, patients with cachexia owing to chronic HF have decreased bone mineral content (112). Whether exercise training (cardiac rehabilitation) of patients with HF can improve osteokine levels awaits to be determined.

### Senolytics

Cellular senescence induces the senescence-associated secretory phenotype, which causes chronic inflammation and contributes to the acceleration of aging of an individual. Recently, senolytic drugs have been developed to remove senescent cells; a senolytic cocktail (dasatinib and quercetin) decreases naturally occurring senescent cells and their secretion of frailty-associated proinflammatory cytokines in human adipose tissues (113). It was also shown that such senolytics can enhance healthspan, as well as lifespan, of aged mice (113).

### A Blood Cell Biomarker to Assess Skeletal/Cardiac Muscle Abnormalities

The mitochondrial function of monocytes, a peripheral blood mononuclear cell (PBMC), provides a possible method to assess skeletal and cardiac mitochondrial function (114). We also reported that the mitochondrial function of PBMCs is decreased in patients with HF, and is associated with disease severity and increased mitochondrial ROS (115). Intriguingly, we moreover demonstrated that mitochondrial ROS levels of PBMCs are closely associated with systemic exercise tolerance (115).

## Conclusion and Future Perspectives

In this review, we discussed the pathology of skeletal muscle abnormalities in HF, including fat accumulation and dysfunction of adipose tissues. Recent advancements in this research field have demonstrated an inseparable association between skeletal muscle abnormalities and adipose tissues of animals with HF, in which these abnormalities can be both the causes and results of HF. Thus, these abnormalities can be therapeutic targets to treat HF, because skeletal muscle and adipose tissue are readily used for clinical testing and diagnoses, as well as for therapeutic interventions; whereas cardiac muscle are difficult to be engineered (Figure 1).

On the other hand, our understanding of the precise molecular and biochemical association between these abnormalities and HF remains incomplete. In particular, given that skeletal muscle abnormalities are frequent in the chronic phase of HF and that such abnormalities are crucial to the worsening of HF, it is very important to identify biomarkers that predict the onset of muscle abnormalities in HF, as well as the molecular mechanisms involved therein. Moreover, as we have discussed above, sarcopenia is also closely associated with HF, with regard to abnormalities of the skeletal muscle and the adipose tissue. Again, there remain a lot to be clarified regarding the molecular and biochemical bases of the association between skeletal/cardiac muscle abnormalities and HF (Figure 1).

Lastly, adequate levels of daily physical exercise are very effective for preventing HF, and are also effective for the treatment of HF. Not only does physical exercise enhance systemic blood circulation and lymphatic flow, which are both crucial for individual immunity, but physical exercise produces myokines in the peripheral blood stream that improve the condition of skeletal muscle mitochondria and maybe also the nervous system, as stated earlier. Analysis of skeletal muscle biopsies has shown that exercise therapy is effective in curing HF with preserved EF patients by repairing skeletal muscle abnormalities and improving exercise tolerance (116–122). A deeper and more precise molecular understanding of our body and its basic design, which has been formed during our evolution on this planet, namely, “HUMAN BIOLOGY,” will help toward developing “natural” methods with minimal medical interventions, rather than developing conservative drugs as HF therapeutics, as well as for the prevention of HF.

## Author Contributions

ST, HS, and SK wrote the manuscript.

## Conflict of Interest

The authors declare that the research was conducted in the absence of any commercial or financial relationships that could be construed as a potential conflict of interest.
